# Improving nitrogen source utilization from defatted soybean meal for nisin production by enhancing proteolytic function of *Lactococcus lactis* F44

**DOI:** 10.1038/s41598-017-06537-w

**Published:** 2017-07-21

**Authors:** Jiaheng Liu, Jianjian Zhou, Lihong Wang, Zelin Ma, Guangrong Zhao, Zhiqiang Ge, Hongji Zhu, Jianjun Qiao

**Affiliations:** 1 Key Laboratory of Systems Bioengineering, Ministry of Education (Tianjin University), Tianjin, 300072 China; 2 School of Chemical Engineering and Technology, Tianjin University, Tianjin, 300072 China; 3 Collaborative Innovation Center of Chemical Science and Engineering (Tianjin), Tianjin, 300072 China

## Abstract

Nisin, one kind of natural antimicrobial peptide, is produced by certain *Lactococcus lactis* strains, which generally require expensive high-quality nitrogen sources due to limited ability of amino acids biosynthesis. Here we use defatted soybean meal (DSM) as sole nitrogen source to support *L*. *lactis* growth and nisin production. DSM medium composition and fermentation conditions were optimized using the methods of Plackett-Burman design and central composite design. The highest nisin production of 3879.58 IU/ml was obtained in DSM medium, which was 21.3% higher than that of commercial medium. To further increase the utilization ability of nitrogen sources, we enhanced the proteolytic function in *L*. *lactis* through rationally expressing the related enzymes, which were selected according to the compositions of amino acids and molecular weight of peptides in DSM medium. Significantly, an artificial proteolytic system consisting of a heterologous protease (NprB), an oligopeptides transporter subunit (OppA) and two peptidases (PepF and PepM) was introduced into *L*.*lactis*. The constructed strain BAFM was capable of achieving efficient biomass accumulation and nisin yield with 30% decreased amount of DSM hydrolysates, which further reduced the cost of nisin production. The strategy described here offers opportunities for low-cost *L*. *lactis* fermentation and large-scale nisin production in industry.

## Introduction

Nisin, one kind of 34-amino-acid-long natural antimicrobial peptide, could effectively inhibit pathogenic and spoilage microorganisms in food without resistance and allergic reactions^1, 2^. It has been widely used as a safe and effective food preservative and a potential agent in veterinary and pharmaceutical products^3–5^. In industry, nisin is produced by certain strains of *Lactococcus lactis* ^6–8^. *L*. *lactis* is nutritionally fastidious microorganism due to the lack of many metabolic pathways, especially the biosynthetic pathways of amino acids^9^. Therefore, *L*. *lactis* generally requires large amounts of expensive high-quality nitrogen sources such as tryptone, peptone, yeast extract, corn steep liquor or meat extract. In most cases, these nitrogen sources must be incorporated as a formulation, which dramatically increased the costs of large-scale nisin production^10, 11^. Therefore, many studies have focused on searching for possible alternatives such as fishery residues^12, 13^, muscle processing wastes^14^ and agricultural wastes^15–17^ to reduce the cost of *L*. *lactis* fermentation and nisin production.

Soybean is a renewable and inexpensive agricultural feedstock, which contains approximately 40% proteins^18, 19^. It has been commercially applied in extracting soy oil, a kind of nutrient-rich and healthy plant oil. Defatted soybean meal (DSM), an abundant byproduct in soy oil industry, has been widely used as animal diets, especially for poultry and swine^20^. Due to its high consistency, nutrient availability, desirable amino acid content and low cost^21–23^, DSM has great potential to serve as nitrogen source for *L*. *lactis* fermentation. However, the nitrogen in soybean meal is mainly macromolecule proteins which need to be further degraded into small molecule peptides and even amino acids before being absorbed and utilized by *L*. *lactis*. Therefore hydrolysis processes including enzymatic hydrolysis and chemical hydrolysis are required to attain absorbable amino acids and peptides. Compared to chemical hydrolysis, enzymatic hydrolysis generally proceeds under moderate conditions and generates few undesirable products. Furthermore, the functionality of the hydrolysis products can be controlled through specific enzymes selection and reaction factors^24^.

However, it is unlikely to realize complete degradation of macromolecule proteins in DSM using commercial protease even after a severe pretreatment. Indeed, the problem of inefficient absorption and utilization is also encountered by commercial nitrogen sources such as peptone and yeast extract, which are generally excessive and remain large amount of residues in *L*. *lactis* fermentation. To address this issue, a new strategy to improve the efficiency of nitrogen source utilization via enhancing proteolytic function was proposed, which could significantly reduce the amount of DSM hydrolysates in the medium.

*L*. *lactis* has intricate machinery for proteolysis which converts exogenous proteins to peptides and then to free amino acids. The proteolytic system of *L*. *lactis* comprises three major components: a cell-envelope proteinase (CEP), amino acid and peptide transport systems and a multitude of peptidases^25^. The exogenous proteins are first hydrolyzed to peptides by CEP. Then peptide transporters including oligopeptide transporters (Opp) and di-tripeptide transporters (DtpP and DtpT) are responsible for uptake of these peptides into the cell. It has been reported oligopeptide is the most widely used nitrogen type by *L*. *lactis* and Opp is most critical among the transporters^26^. Finally, catalyzed by peptidases, these peptides are completely degraded to free amino acids which are essential for synthesis of key metabolites and growth of *L*. *lactis* strain^27, 28^. In this study, we rationally engineered *L*. *lactis* to increase the utilization ability of DSM based on the molecular weight of peptides and amino acids composition in DSM hydrolysates. First we enhanced the ability of protein hydrolysis, oligopeptides transport and peptides degradation through heterologous expression of extracellular proteases from *Bacillus subtilis* 168, overexpression of Opp related genes and peptidase in *L*. *lactis* F44, respectively. Second, the efficacy of engineered strains was evaluated based on cell growth and nisin production with a decreased amount of DSM hydrolysates. Subsequently, an artificial proteolytic system consisting of the most effective genes was constructed and transferred into *L*. *lactis* F44 to realize more efficient utilization of DSM proteins.

Therefore, the objectives of this research were to: (1) investigate the impacts of enzymatic hydrolysis conditions on (degree of hydrolysis) DH of DSM, (2) develop and optimize a medium using DSM hydrolysates as sole nitrogen source which supports better growth and higher nisin production than commercial medium applied in industry, (3) engineer *L*. *lactis* to enhance its proteolytic function through constructing an artificial proteolytic system and (4) evaluate the effectiveness of the constructed strains on strain growth and nisin production with a decreased amount of DSM hydrolysates.

## Results

### Effect of enzymatic hydrolysis on DH of DSM

**Figure 1:**
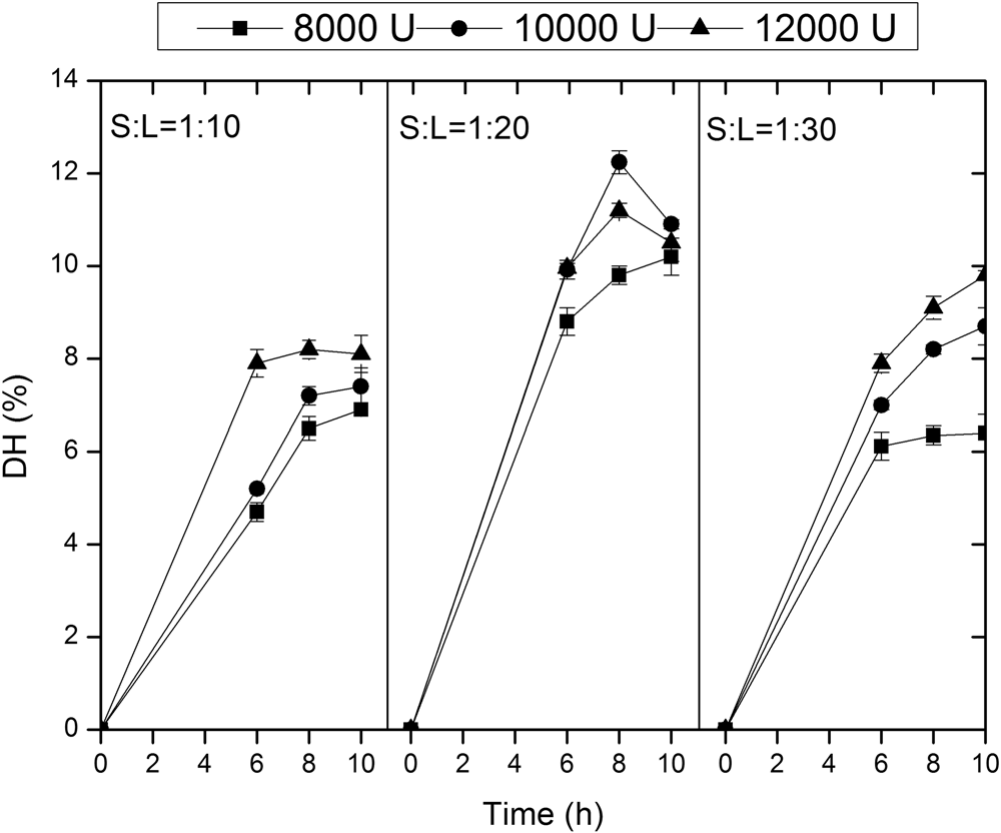
The DH value of DSM presented as a function of enzyme loading, hydrolysis time and S:L. The enzymatic hydrolysis was conducted at 50 °C with constant agitation (100 rpm orbital shaking). Average data of triplicate experiments were presented. Error bars represent standard deviations from three parallel replicates.

Twenty-seven enzymatic hydrolysis combinations derived from varying parameters including enzyme loading (U/g, 3 levels), hydrolysis time (h, 3 levels) and solid/liquid (S/L, g/ml, 3 levels), were conducted in triplicate on DSM. As shown in Fig. 1, under all the combined enzyme loading and hydrolysis time conditions, DH values attained with a S:L of 1:20 were significantly higher than that of 1:10 and 1:30. When S:L was 1:20 and 1:30, elevation in enzyme loading and hydrolysis time both increased DH value obviously indicating an increased demand for enzyme dosage and hydrolysis time. Enzymatic hydrolysis with enzyme loading of 12000 U/g for 10 h attained the highest DH value of 9.87% when S:L was 1:30. Similar DH value in response to enzyme loading and enzymatic time had been reported by Kong *et al*.^29^. When S:L was 1:20, it was shown that enzyme loading of 10000 U/g and 12000 U/g gave almost same DH value for 6 h which was higher than that with enzyme loading of 8000 U/g. Using 10000 U/g enzyme loading, the DH value increased marginally with increase of hydrolysis time to 8 h and then decreased obviously at 10 h. The highest DH value of 12.24% could be attained with enzyme loading of 10000 U/g for 8 h and S:L 1:20.

### Statistical optimization of DSM medium compositions and fermentation conditions for nisin production

#### Plackett-Burman design (PBD)

A 12 run PBD^30, 31^ was explored for screening variables based on our previous research^32^. Each independent variable with high and low levels, denoted by (+) and (−) respectively, was evaluated. In this study, 8 assigned variables (DSM hydrolysates, sucrose, KH_2_PO_4_, cysteine, NaCl, MgSO_4_·7H_2_O, pH and inoculum amount) were chosen for optimization and the response was measured in terms of nisin production. The symbols, units and actual levels of the variables were shown in Table 1.

**Table 1:** Variables and their levels chosen for the experimental design.

Factor	Units	Symbols	Coded levels		
			−1	0	+1
DSM hydrolysates	g/L	X_1_	25	30	35
Sucrose	g/L	X_2_	9	12	15
KH_2_PO_4_	g/L	X_3_	15	20	25
Cysteine	g/L	X_4_	0.15	0.20	0.25
NaCl	g/L	X_5_	1.0	1.5	2.0
MgSO_4_·7H_2_O	g/L	X_6_	0	0.05	0.10
pH		X_7_	7	7.3	7.6
Inoculum amount	%, v/v	X_8_	1	3	5

Table 2 showed the design matrix and its corresponding results. The production of nisin varied from 1793 IU/ml to 3751 IU/ml under different levels of factors, which indicated that the parameters of fermentation could directly affect nisin production. The regression analysis of variance (ANOVA) based on the experimental data was shown in Table 3. The coefficient of determination R_2_ of the model was 0.9608, which explained 96.08% of the response variability. The significance of corresponding variable was determined by *p*-values and *p*-values of less than 0.05 were considered to be significant effects on the response. As shown in Table 3, the *p*-value of X_3_ and X_8_ was 0.029 and 0.013, respectively. Both of them were less than 0.05, indicating that the concentration of KH_2_PO_4_ (X_3_) and inoculum amount (X_8_) played significant role in nisin production. The variables with insignificant effects among the factors in this study were not included in the next optimization step.

**Table 2:** Plactkett-Burman design variables (in code levels) with nisin titer as response (8 h anaerobic fermentation at 30 °C).

Run	X_1_	X_2_	X_3_	X_4_	X_5_	X_6_	X_7_	X_8_	Nisin titer (IU/ml)*
1	1	−1	1	1	−1	1	1	1	2610.54 ± 78.64
2	−1	1	1	0	1	1	1	−1	2308.45 ± 80.56
3	1	1	0	0	−1	1	−1	1	3263.92 ± 123.11
4	−1	−1	0	0	−1	−1	−1	−1	1793.80 ± 23.56
5	−1	1	0	1	1	−1	1	1	2539.45 ± 63.56
6	1	1	1	0	−1	−1	1	−1	3751.76 ± 231.63
7	−1	−1	0	1	−1	1	1	−1	2317.15 ± 134.00
8	1	−1	1	1	1	−1	−1	−1	2670.18 ± 57.89
9	1	1	0	1	1	1	−1	−1	2325.89 ± 89.00
10	−1	−1	1	0	1	1	−1	1	2765.67 ± 99.23
11	1	−1	0	0	1	−1	1	1	3263.92 ± 189.55
12	−1	1	1	1	−1	−1	−1	1	2565.07 ± 135.11

*Values were given by mean ± standard deviation (n = 3).

**Table 3:** ANOVA for the Plactkett-Burman factorial model.

Source	Sum of Squares	Degree of Freedom	Mean of Square	F-Value	*p*-value
Model	3510739	8	438842	9.20	0.047
X_1_	247411	1	247411	5.19	0.107
X_2_	264218	1	264218	5.54	0.100
X_3_	738745	1	738745	15.49	0.029
X_4_	36754	1	36754	0.77	0.445
X_5_	195624	1	195624	4.10	0.136
X_6_	346689	1	346689	7.27	0.074
X_7_	297207	1	297207	6.23	0.088
X_8_	1384091	1	1384091	29.02	0.013
Residual	143066	3			
Cor Total	3653805	11			

R^2^ = 0.9608, Adj R^2^ = 0.8564.

#### Central composite design (CCD)

**Figure 2:**
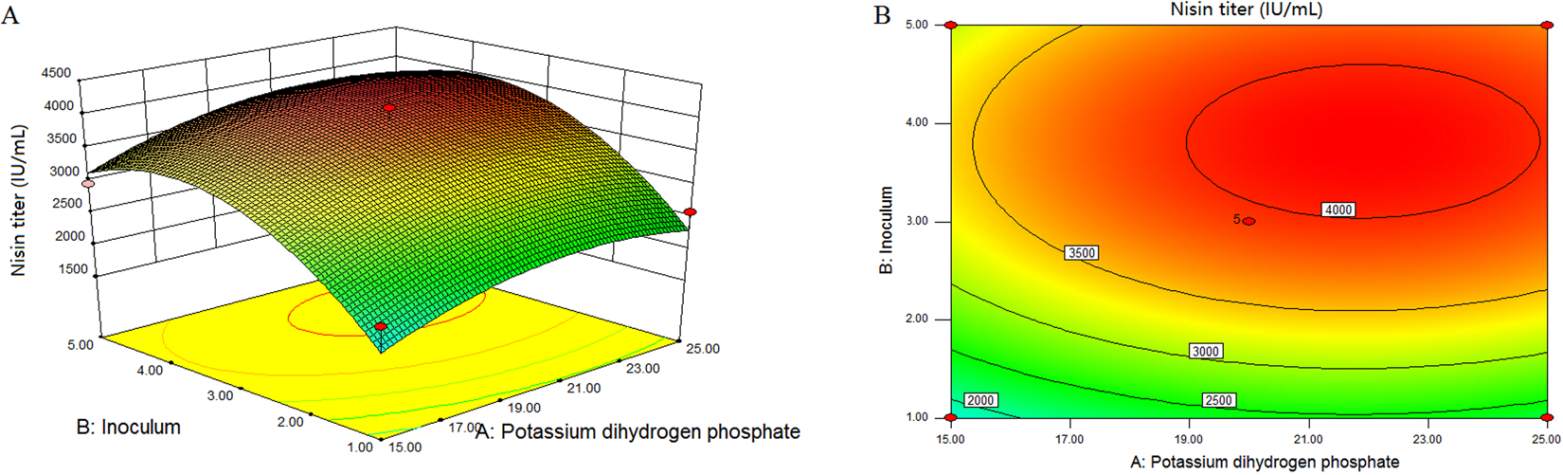
(**A**) Response surface plot and (**B**) corresponding contour of the mutual effects of KH_2_PO_4_ and inoculum amount on nisin titer (8 h anaerobic fermentation at 30 °C).

The experimental design and results of CCD were presented in Table 4. The regression analysis of a full second-order polynomial model was shown in Table 5. The F-value of the model was 20.46, which implied that the model was so significant that there was only 0.05% chance for the model to occur due to noise. Additionally, with a relatively high value of the determination coefficient (R_2_ = 0.9359), the model fitted to experimental results very well that provides a good estimation of nisin yield. The term was regarded as significant with a *p*-value less than 0.05. In this case, the linear and quadratic of X_3_, X_8_, X_3_^2^, X_8_^2^ (p < 0.05) were recognized as the significant model terms, suggesting that K_2_HPO_4_ and inoculum amount play a significant role on nisin production by culture of *L*. *lactis* F44, However, their interaction between K_2_HPO_4_ and inoculum amount (X_3_X_8_, *p* > 0.05) was insignificant on nisin production. The quadratic equation illustrates the relationship between KH_2_PO_4_ (X_3_) and inoculum amount (X_8_) corresponding to nisin yield (Y) as follows:

1 $$Y=3936.67+273.07{X}_{3}+683.9{X}_{8}+17.99{X}_{3}{X}_{8}-367.66{X}_{3}^{2}-842.23{X}_{8}^{2}$$

where Y was the predicted nisin yield, X_3_ and X_8_ were the coded values of K_2_HPO_4_ and inoculum amount. The regression model (Eq. 1) can be used to predict the range of nisin production for various levels of the selected variables. According to calculation, the maximum production of nisin (Y) was 3988.32 IU/ml when the optimal concentration of KH_2_PO_4_ (X_3_) and inoculum amount (X_8_) was 25 g/l and 3.83%, respectively (Fig. 2).

**Table 4:** Experimental design and the results of CCD.

Run	K_2_HPO_4_ (X_3_) (g/l)	Inoculum amount (X_8_) (%,V/V)	Nisin titer (IU/ml)	
			Observed*	Predicted
1	20	5.82843	3533.38 ± 134.78	3218.05
2	20	3	3827.47 ± 231.00	3936.67
3	20	3	3917.46 ± 152.61	3936.67
4	27.0711	3	3589.76 ± 98.77	3598.26
5	25	5	3451.22 ± 56.12	3701.67
6	20	3	4119.35 ± 110.85	3936.67
7	25	1	2582.35 ± 78.99	2297.89
8	15	1	2163.89 ± 34.98	1787.87
9	15	5	2960.81 ± 145.00	3119.69
10	20	3	3902.14 ± 210.19	3936.67
11	20	0.17157	841.905 ± 15.64	1282.62
12	12.9289	3	2687.81 ± 73.78	2807.66
13	20	3	3916.85 ± 45.21	3936.67
Max	25	3.83	3879.58 ± 340.07	3988.24

*Values were given by mean ± standard deviation (n = 3).

**Table 5:** Regression analysis of a full second-order polynomial model for optimization of nisin production.

Source	Sum of Squares	df	Mean Square	F-value	*p*-value Prob > F
Model	9.75E + 06	5	1.95E + 06	20.46	0.0005
X_3_	5.96E + 05	1	5.96E + 05	6.25	0.0409
X_8_	3.74E + 06	1	3.74E + 06	39.26	0.0004
X_3_X_8_	1294.27	1	1294.27	0.014	0.9105
X_3_X_3_	9.40E + 05	1	9.40E + 05	9.86	0.0164
X_8_ X_8_	4.94E + 06	1	4.94E + 06	51.84	0.0002
Residual	6.68E + 05	7	95358.18		
Lack of Fit	6.20E + 05	3	2.07E + 05	17.5	0.0092
Pure Error	47250.69	4	11812.67		
Cor Total	1.04E + 07	12			

R^2^ = 0.9359; Adj R^2^ = 0.8902; C.V.% = 9.67; Adeq Precision = 12.651.

### Experimental validation of the optimized medium and fermentation conditions

**Figure 3:**
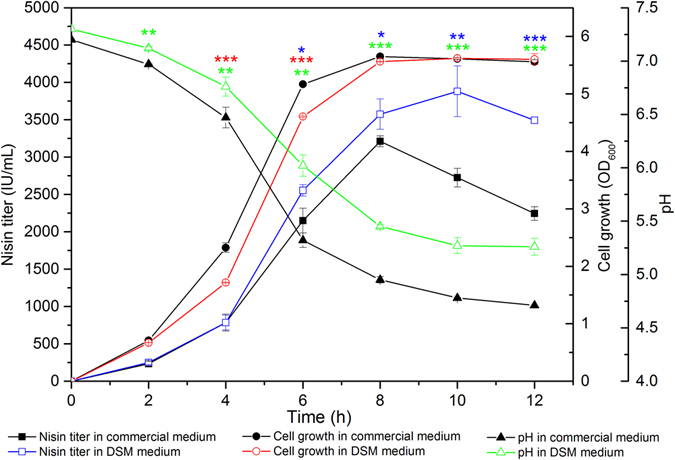
Time profile of nisin titer (square), cell density (circle) and pH (triangle) of *L*. *lactis* F44 cultured in DSM medium (hollow symbols) and commercial medium (solid symbols). Average data of triplicate experiments were presented. Error bars represent standard deviations from three parallel replicates. The data were analyzed by One-way ANOVA. Statistically significant differences between DSM medium groups and commercial medium groups were indicated by for *p < 0.05 for **p < 0.01 and for ***p < 0.001.

To verify the model adequacy and investigate the effect of nitrogen sources on nisin production, fermentation experiments were conducted using DSM medium under the conditions optimized by the methods of PBD and CCD. And the commercial medium described in “Methods” was used for comparison. Figure 3 showed nisin production, cell growth and pH of fermentation broth of *L*. *lactis* F44 in optimized DSM medium and commercial medium respectively. In DSM medium, the maximum production of nisin was 3879.58 IU/ml at 10 h, which agrees excellently with the predicted value (3988.32 IU/ml). Significantly, the nisin production attained in DSM medium was 21.25% higher than that in the commercial fermentation medium (3199.57 IU/ml), which indicated that DSM could be proper nitrogen source for *L*. *lactis*. F44 showed a lower growth rate before 8 h in DSM medium than that in commercial medium presumably due to the typical inhibitors from DSM hydrolysates. An identical cell density was attained at 10 h in DSM and commercial medium. In addition, obvious difference of pH variation was observed between DSM and commercial medium. Fermentation in DSM medium exhibited a higher final pH and lower decreasing rate of pH than that in commercial medium. There might be some buffer substances in DSM medium. Since acidic environment had a detrimental effect on *L*. *lactis* growth, this could benefit nisin production.

### Compositions of free amino acids and molecular weight distribution of peptides in DSM hydrolysates

Concentrations of free amino acids detected in DSM hydrolysates were shown in Table 6. Histidine, methionine, leucine, isoleucine, valine and arginine have been identified as essential amino acids for *L*. *lactis* in a previous report^33^. Thus, DSM hydrolysates might be very beneficial for fermentation of *L*. *lactis* F44 due to its higher content of these amino acids. In addition, there were lower content of some amino acids such as lysine, glutamate, serine and methionine in DSM hydrolysates. However, it has been reported that these amino acids were abundant in soybean and DSM^34^. This indicated that these amino acids might mainly exist in the form of protein and peptides.

**Table 6:** Free amino acid composition in DSM hydrolysates.

Amino acids	Composition (ng/mL)*
Glycine	2907.21 ± 164.48
Alanine	1522.00 ± 53.79
Valine	1970.56 ± 68.47
Leucine	2660.30 ± 123.59
Isoleucine	1396.68 ± 46.79
Phenylalanine	1678.96 ± 63.13
Proline	2228.12 ± 75.75
Serine	958.19 ± 29.86
Tyrosine	1651.01 ± 126.67
Methionine	878.06 ± 34.15
Threonine	1819.96 ± 45.15
Aspartate	1205.25 ± 35.69
Glutamate	610.96 ± 16.35
Lysine	14.23 ± 63.58
Arginine	3064.82 ± 166.75
Histidine	1013.97 ± 87.77

*Values were given by mean ± standard (n = 3).

The availability of peptides was highly correlated to their molecular weights and peptides with higher molecular weight are difficult for *L*. *lactis* uptake. As shown in Table 7, almost the molecular weight of all peptides were above 4400 Da, which indicated that further degradation of these peptides was needed through proteolytic system in *L*. *lactis*. The peptides in the molecular weight range of 4420–8360 Da accounted for approximately 29.33%, which could be directly transported into the cells by Opp system. The peptides with the molecular weight of above 20000 Da might be the soluble proteins which could hardly be utilized by the strain. Further investigation was in progress to recycle these proteins.

**Table 7:** Molecular weight distribution of peptides in DSM hydrolysates.

Molecular weight (Da)	4420–8360	8390–11860	11870–15151	15155–20175	17935–20175	20176–22820
Content (%)*	29.33 ± 3.58	6.96 ± 1.22	14.36 ± 2.23	14.16 ± 1.08	14.01 ± 2.11	21.13 ± 3.46

*Values were given by mean ± standard (n = 3).

### Selection of genes for enhancing proteolytic function of *L*. *lactis* F44

**Figure 4:**
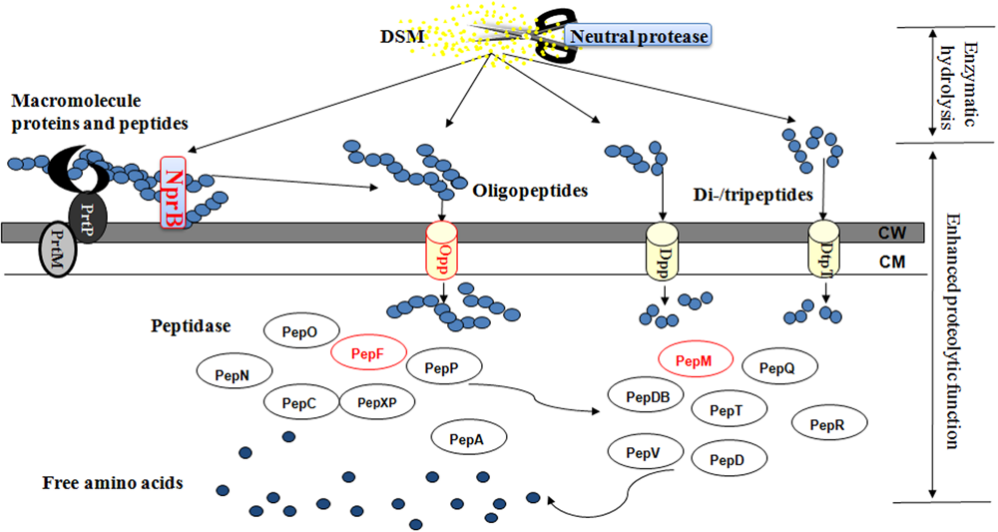
Schematic overview of DSM protein utilization through the proteolytic system in *L*. *lactis* and the genes or components with enhanced activity implemented in this study (red symbols). NprB was a heterologous protease from *B*. *subtilis* 168. The function of Opp system was enhanced by overexpression of OppA, a membrane lipoprotein. Peptides degradation ability was enhanced by overexpression of two peptidases, PepF and PepM. CW: cell wall. CM: cell membrane. CEP: cell-envelope proteinase.

The proteolytic system in *L*. *lactis* was responsible for the utilization of exogenous proteins as the nitrogen source. Hence, to enhance proteolytic function of *L*. *lactis* F44, we increased the expression level of genes encoding key enzymes or components of this system (Fig. 4). The efficacy of engineered strains was evaluated based on cell growth and nisin production with a decreased amount of DSM hydrolysates.

**Figure 5:**
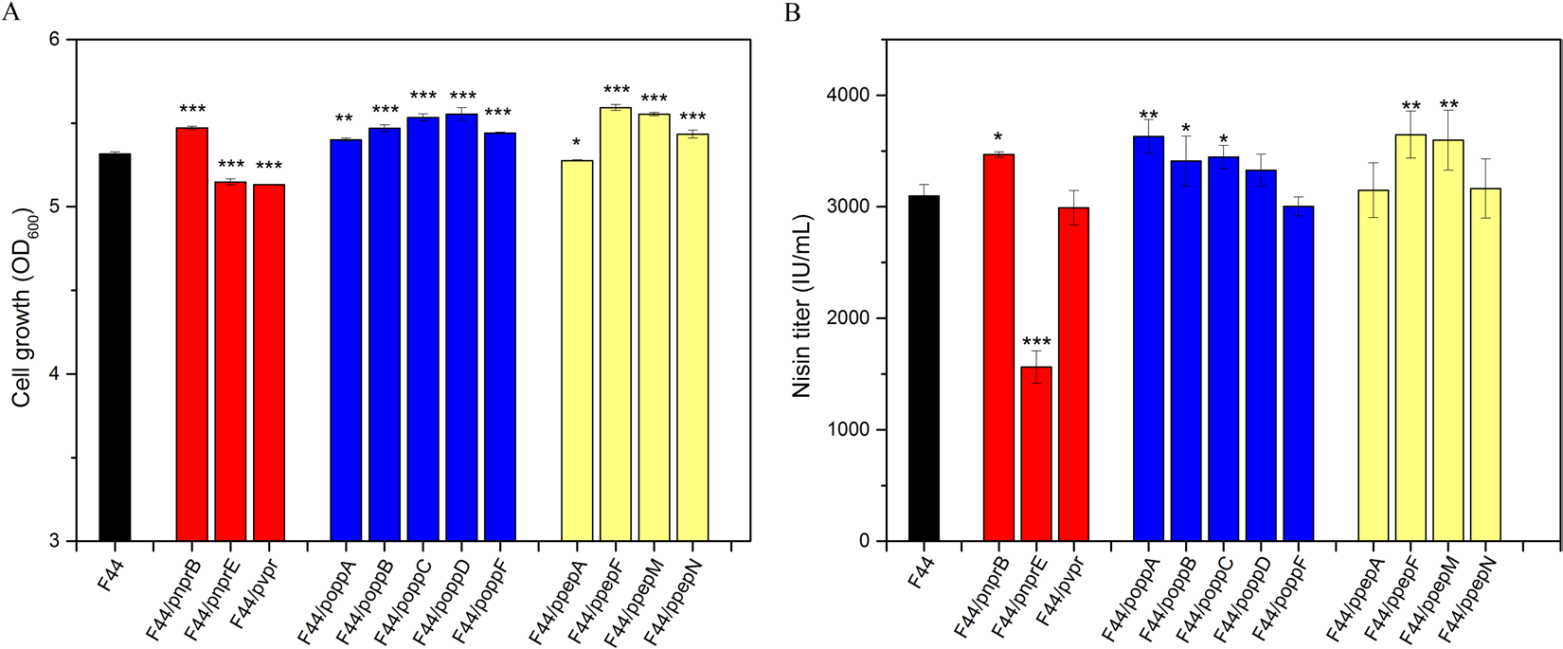
(**A**) Cell density and (**B**) nisin titer of the *L*. *lactis* F44 and engineered strains cultured in DSM medium with 25 g/L DSM hydrolysates (8 h anaerobic fermentation at 30 °C). Average data of triplicate experiments were presented. Error bars represent standard deviations from three parallel replicates. The data were analyzed by One-way ANOVA. Statistically significant differences between engineered strain groups and F44 groups were indicated by for *p < 0.05 for **p < 0.01 and for ***p < 0.001.

As only free amino acids and peptides with lower molecular weight can be directly transported into the cell, we hypothesized that increase in extracellular protease activity of *L*. *lactis* could effectively contribute to the utilization of macromolecular proteins and peptides in DSM medium. Therefore, we cloned *nprB*, *nprE* and *vpr* gene encoding neutral protease, bacillolysin, minor extracellular protease respectively from *B*. *subtilis* 168 into plasmid pLEB124. The constructed plasmids were transformed into *L*. *lactis* F44 to generate strain F44/pnprB, F44/pnprE and F44/pvpr. Then fermentation of the engineered strains was conducted in DSM medium with a decreased amount of DSM hydrolysates (25 g/L) and F44 was also used for comparison. As shown in Fig. 5, introducing *nprB* into F44 significantly increased cell density and nisin production presumably due to the enhanced degradation ability of extracellular protein. However, strain F44/pnprE and F44/pvpr exhibited lower cell density which indicated that bacillolysin and minor extracellular protease might exert inhibition effects on the growth of *L*. *lactis* strain. Interestingly, nisin production dramatically decreased for F44/pnprE. Since nisin was a 34-amino-acid-long natural antimicrobial peptide, it might be degraded by bacillolysin encoded by *nprE* which had a detrimental effect on nisin accumulation.

To increase the utilization of oligopeptides in DSM medium, the Opp related genes including *oppA*, *oppB*, *oppC*, *oppD* and *oppF* were overexpressed respectively in *L*. *lactis* F44 generating strain F44/poppA, F44/poppB, F44/poppC, F44/poppD and F44/poppF. Then we conducted fermentation of these strains in DSM medium with 25 g/L DSM hydrolysates. Figure 5 showed that the maximum nisin production of 3629.16 IU/mL was attained by F44/poppA, which was 17.15% higher than that of F44. F44/poppB, F44/poppC and F44/poppD could also increase the nisin production to varying degrees. Neither nisin production nor strain growth was improved in *oppF* overexpressing strain F44/poppF.

Since the content of some amino acids such as lysine, glutamate, serine and methionine in DSM hydrolysates was very low, it is necessary to increase the supply of these amino acids. One possible strategy for resolving this issue was to enhance the activity of some specific peptidases to further degrade the peptides which were rich in these amino acids. PepN was an aminopeptidase with high specificity to lysine and methionine. As a glutamyl aminopeptidase, PepA played a key role in releasing N-terminal acidic amino acid residues including glutamate and aspartate from decapeptide. PepM, a methionyl aminopeptidase, could cleave N-terminal methionine from proteins^35^. Therefore, PepN, PepA and PepM were overexpressed respectively in F44. In addition, it has been reported that PepF, which could hydrolyze oligopeptides containing 7 to 17 amino acids residues, and contribute to protein turnover under nitrogen limiting conditions^36^. Thus, we also overexpressed PepF in F44. As expected, with a lower level of DSM hydrolysates addition (25 g/L), overexpression of pepM and pepF resulted in an increased nisin titre of 3596.21 IU/mL and 3646.57 IU/mL, representing a 16.09% and 17.71% increase compared with F44, respectively. The cell density achieved by F44/ppepF and F44/ppepM were obviously higher than that of F44.

### Constructing an artificial proteolytic system in *L*. *lactis* F44

**Figure 6:**
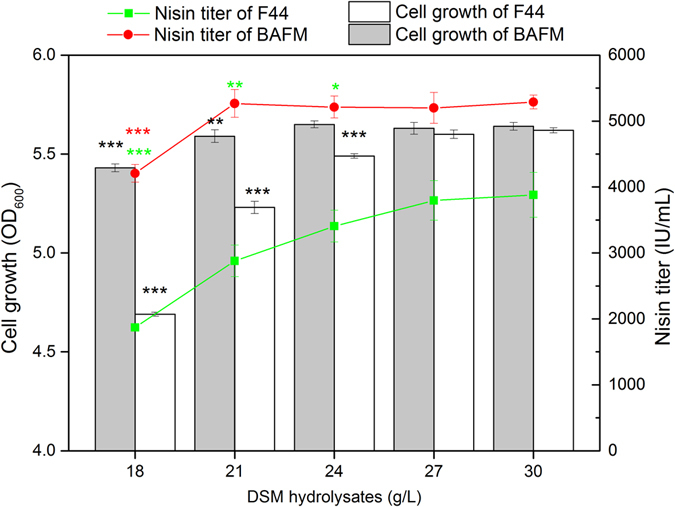
Effect of DSM hydrolysates amount on cell growth and nisin titer of F44 and BAFM. Average data of triplicate experiments were presented. Error bars represent standard deviations from three parallel replicates. The data were analyzed by One-way ANOVA. Statistically significant differences between engineered strain groups and F44 groups were indicated by for *p < 0.05 for **p < 0.01 and for ***p < 0.001.

Since the heterologous expression of protease NprB and overexpression of oligopeptides transporter subunit (OppA) and two peptidases (PepF and PepM) could significantly increase the ability of nitrogen utilization in DSM medium, we constructed an artificial proteolytic system through combined expression of tetrad genes, *nprB*, *oppA*, *pepF* and *pepM*. The highest nisin titer of the engineered strain BAFM was up to 5288.89 IU/mL, increased by 36.33% compared with F44 strain in DSM medium (30 g/L DSM hydrolysates). Despite the increased nisin production by BAFM, enhancing proteolytic function had the potential to further reduce the demand for nitrogen source in medium, which could significantly reduce the cost of fermentation process. Therefore, the addition of DSM hydrolysates concentration in DSM medium was decreased from 30 g/L to 27, 24, 21, 18 g/L respectively and their effects on strain growth and nisin producution of F44 and BAFM were investigated to provide comparative performance data. As shown in Fig. 6, 21 g/L DSM hydrolysates was adequate to support an equivalent cell density and nisin yield to 30 g/L DSM hydrolysates for BAFM. However, the strain growth and nisin production of F44 were obviously decreased with 24 g/L DSM hydrolysates compared to that with 30 g/L. These results demonstrated that the demand for DSM hydrolysates by BAFM was significantly reduced and 30% of DSM hydrolysates could be saved. Thus the cost of nisin production could be further reduced. Thus, shake-flask fermentation of BAFM in DSM medium with 21 g/L DSM hydrolysates was performed and nisin production, cell density and pH value were monitored. Meanwhile, fermentation in commercial medium was also conducted to provide comparative performance data (Supplementary Fig. S1). In DSM medium, the maximum nisin titer was 5267.13 IU/mL at 10 h which was significantly higher than that in commercial medium. BAFM showed an obviously increased cell density and nisin production in DSM medium than that of F44. However, similar cell density and nisin production were attained by F44 and BAFM in commercial medium (Fig. 3, Supplementary Fig. S1). This was presumably due to the sufficient and effective nitrogen sources in commercial medium and further proved the abundant necessity and feasibility of enhancing proteolytic function towards efficient utilization of nitrogen sources in DSM hydrolysates.

## Discussion

Nisin production and application have received widespread attention from researchers in academic and industry fields. Many strategies have been applied to increase nisin yield and cut productive costs, such as optimization of fermentation process^37, 38^, application of cost-effective medium^39, 40^ and gene engineering of strains^32, 41^. In this study, combined strategy of DSM medium development and strains improvement was applied to enhance nisin Z production and reduce production costs.

Due to its limited capacity to synthesize amino acids, fermentation of *L*. *lactis* strains was very fastidious which required large amounts of expensive nitrogen sources such as peptone, tryptone, yeast extract, meat extract and even their formulation. This dramatically increased the costs of large-scale nisin production. DSM, an abundant byproduct in soy oil industry, has great potential as cheap nitrogen source for *L*. *lactis* fermentation due to its high protein content, rich content of essential amino acids and low price. However, degradation of DSM proteins into small peptides and free amino acids is required to promote *L*. *lactis* utilization.

The results of enzymatic hydrolysis of DSM were showed in Fig. 1. DH was used in this study to evaluate the hydrolysis effectiveness of DSM. The enzyme used in this research was a commercial neutral protease which has been widely applied in enzymatic hydrolysis of soy protein due to its proven effectiveness and neutral hydrolysis condition. The maximum DH value we reported was close to that of other researches such as enzymatic hydrolysis of defatted soy flour^24^ and soy proteins^42^. The slight difference was probably owing to the different physical compositions and structures of feedstocks and protease types.

PB design was used to identify variables that had significant effects on nisin production. The experimental design and results of PBD were presented in Table 2, the concentration of KH_2_PO_4_ and inoculum amount played significant role in nisin production. The phosphorus source KH_2_PO_4_ has been reported to be significant in nisin production^43, 44^. Moreover, KH_2_PO_4_ could act as a buffer for the broth as a salt of weak acid. Then CCD experiments were conducted. Three dimensional response surface and corresponding contour of K_2_HPO_4_ and inoculum amount were presented in Fig. 2. It was obviously observed that the nisin production increased and then maintained at a high level following the increase of the concentration of KH_2_PO_4_ in the medium. The inoculum amount also had similar effect on nisin production. In summary, Response surface method is an efficient tool to optimize the medium composition for nisin production by F44. Therefore, the optimized medium contained 30 g/l of DSM hydrolysates, 25 g/l of KH_2_PO_4_, 12 g/l of sucrose, 1.5 g/l of NaCl, 0.05 g/l of MgSO_4_·7H_2_O and 0.2 g/l of cysteine.

Fermentation experiments were conducted using DSM medium under the conditions optimized by the methods of PBD and CCD. As shown in Fig. 3, the nisin production attained in DSM medium was 21.3% higher than that in the commercial fermentation medium. This result indicated that DSM hydrolysates could be a proper nitrogen source for *L*. *lactis* fermentation and nisin production. Obviously, the nisin production peaked at 10 h in DSM medium, however, it happened at 8 h in the commercial fermentation medium. This difference was probably due to the DSM oligosaccharides such as stachyose and raffinose. These oligosaccharides could be hydrolyzed to galactose by α-galactosidase which was produced by *L*. *lactis*. It was reported that the changes on pathway of carbon metabolism of *L*. *lactis* under acid stress resulted in an increasing utilization of galactose^45, 46^. Therefore the strain in DSM medium which contained oligosaccharides could maintain better activity than that in the commercial fermentation medium with sucrose as sole carbon source. Further studies needed to be done to determine the effects of these oligosaccharides in DSM medium on strain growth and nisin production.

Analysis of the molecular weight distribution of peptides in DSM hydrolysates demonstrated an incomplete degradation of DSM proteins. Peptides with the molecular weight of above 20000 Da accounted for 21.13%, which could not be directly utilized by the strain in the fermentation process unless they were further hydrolyzed. In addition, some amino acids such as lysine, glutamate, serine and methionine in DSM hydrolysates might be insufficient for *L*. *lactis* growth due to their low contents. Indeed, inefficient absorption and utilization is also encountered by commercial nitrogen sources such as peptone and yeast extract, which resulted in an excess addition and waste of these resources in industry. To address these issues, a new strategy for improving the utilization efficiency of nitrogen source through enhancing proteolytic function was proposed to save DSM hydrolysates. The efficacy of engineered strains was evaluated based on cell growth and nisin production with a decreased amount of DSM hydrolysates.

*L*. *lactis* possesses a complex proteolytic system, which converts extracellular nitrogen sources to amino acids for maintaining its metabolism and growth (Fig. 4). The first step in proteolytic system is degradation of proteins in the medium to oligopeptides by extracellular proteinase such as PrtP^47^. Despite enzymatic hydrolysis by neutral protease, peptides with high molecular weight were abundant in DSM hydrolysates (Table 7). It has been reported that *B*. *subtilis* were prolific producers of extracellular proteases including neutral protease and alkaline protease^48^. Taking the optimal pH of *L*. *lactis* growth into consideration, 3 neutral proteinases (NprE, NprB and Vpr), which were widespread in *B*. *subtilis* and had broad substrate specificity, were introduced into *L*. *lactis* F44, respectively, to further degrade the proteins in DSM hydrolysates. The higher biomass accumulation and nisin production achieved by F44/pnprB with decreased amount of DSM hydrolysates indicated that the extracellular protease encoded by *nprB* significantly contributed to hydrolysis of proteins from DSM hydrolysates. Although NprE and Vpr have been proved to be effective in *B*. *subtilis*, these proteases might have poor specificity to degrade DSM proteins. The engineered strain *L*. *lactis* F44/pnprE even reduced nisin production suggesting that the protease nprE, also known as bacillolysin, might degrade nisin, a 34-amino-acid-long natural antimicrobial peptide. Therefore, to select more extracellular proteases, it was important to consider the following criteria: (i) high specificity to DSM proteins, (ii) high efficiency under neutral or weak acidity conditions, (iii) no inhibitory effects on strain growth and (iv) no degradation of nisin.

In the second step, peptide transporters including Opp, DtpP and DtpT were responsible for uptake of extracellular peptides into the cell. One reason that we overexpressed Opp related genes in this study was that few dipeptides and tripeptides were detected and oligopeptides were the biggest proportion in DSM hydrolysates; on the other hand, it has been reported Opp was the most critical transporter in *L*. *lactis* ^26^. In addition, our recent research found that under some extreme environments including acidic conditions and nutritional deficiency, the Opp related genes *oppA*, *oppB*, *oppC*, *oppD* and *oppF* were notably up-regulated (unpublished data). Thus, there was a need to realize a more efficient transportation of oligopeptides through enhancing the function of Opp system. Collectively, these facts motivated us to enhance oligopeptides transport ability through overexpression of Opp related genes towards a better utilization of DSM peptides and higher yield of nisin. The results of fermentations performed with engineered *L*. *lactis* strains indicated that overexpression of oppA might significantly promote the uptake of DSM peptides, which resulted in higher cell density and nisin production with 25 g/L DSM hydrolysates (Fig. 5). OppA is a membrane lipoprotein which could identify and combine with peptides from external environment. Picon *et al*. reported that *L*. *lactis* strains with the absence of *oppA* could hardly grow on some peptides such as DRVYIHPFHL, it plays a crucial role in peptide utilization^49^. Previous study showed that knock-out of *oppA* resulted in a low efficiency of Leu-enkephalin utilization even with expression of *oppA* (an *oppA* homologue) in *L*. *lactis* MG1363^50^. In addition, F44/poppB, F44/poppC and F44/poppD could also increase the nisin production to varying degrees (Fig. 5B). Therefore, further improvement of nisin production in DSM medium was possible, if *oppA*, *oppB*, *oppC* and *oppD* were simultaneously overexpressed. Although oppD and oppF are both ATP-binding protein, neither nisin production nor strain growth was improved in *oppF* overexpressing strain F44/poppF, It has been reported that oppD and oppF could couple energy to the transport process independently and were even interchangeable for ATP-binding function. However, both oppD and oppF are required for normal function of Opp system^51^. Thus, it cannot be totally excluded that oppD or oppF serves another role^52^. In this study, oppD might play a crucial role for nutrient acquisition and nisin production in DSM medium. Another possible explanation might be that each of the ATP-binding protein functions as a dimer. Then oppD and oppF formed a homodimer or a large oligomer which functions for the transport system^53, 54^. Thus, we speculated that low expression level of oppD might be the limiting factor for ATP binding ability while oppF not.

The third step in proteolytic system was degradation of intracellularly accumulated peptides catalyzed by multiple peptidases. These peptidases with different substrates specificity could release different amino acids. To compensate for the insufficient amino acids including methionine, glutamate and lysine in DSM medium, pepM, pepA, pepN and pepF were overexressed to increase their acitivity, respectively. The fermentation results indicated that overexpression of pepM and pepF could significantly promote strain growth and nisin production with less supply of DSM hydrolysates (Fig. 5).

To further decrease the quantity of DSM medium demanded by *L*. *lactis* F44, an artificial proteolytic system through co-expression of a heterologous protease (NprB), a oligopeptides transporter subunit (OppA) and two peptidases (PepF and PepF) was constructed and introduced into *L*.*lactis* F44. The engineered strain BAFM was capable of achieving efficient biomass accumulation and nisin yield with 30% decreased amount of DSM hydrolysates (Fig. 5). Thus the cost of nisin production could be further reduced.

In conclusion, this research provided an efficient and economic process for efficient utilization of DSM through combined medium optimization and strain improvement. Further studies are in progress to identify the effective peptides in DSM hydrolysates for *L*. *lactis* fermentation and evaluate the efficacy of these peptides. Although the results demonstrated the production of a natural antimicrobial peptide, nisin, the cost-effective fermentation using DSM as solo nitrogen source could also be extended to produce other value-added bioproducts by *L*. *lactis* such as lactic acid, exopolysaccharides, vitamins and vaccines. In addition, the artificial proteolytic system constructed in this work was of critical importance to make nitrogen substrates more economically feasible, which had the potential to be applied in other amino acid auxotrophous bacteria.

## Materials and Methods

### Strains and growth conditions

The bacterial strains and plasmids used in the study are listed in the Supplementary Table S1. We constructed the engineered strain *L*. *lactis* F44 with a high nisin Z production through genome shuffling of *L*. *lactis* YF11 (accession number CGMCC7.52) in our previous study^32^. The inoculum was prepared in a 250-ml Erlenmeyer flask containing 100 ml of seed medium (w/v) with peptone (1.5%), yeast extract (1.5%), sucrose (1.5%), KH_2_PO_4_ (2.0%), NaCl (0.15%) and MgSO_4_·7H_2_O (0.015%). *Escherichia coli* TG1, used for plasmid preparation, and *B*. *subtilis* 168 were grown at 37°C with shaking at 180 rpm in Luria-Bertani (LB) medium. *Micrococcus flavus* ATCC 10240, preserved in the laboratory, was used as an indicator strain for the bioassay of nisin. M. *flavus* ATCC 10240 was grown on medium containing 0.8% tryptone, 0.5% glucose, 0.5% yeast extract, 0.5% NaCl, 0.2% Na_2_HPO4, and 1.5% agar.

### Enzymatic hydrolysis of DSM

The DSM in this study was obtained from Huayu Co. Ltd. (Qufu, China). After mechanical grinded and dried, the DSM was pretreated 80 °C for 10 min. The enzyme, which was a neutral endopeptidase produced by *B*. *subtilis* with an enzyme activity of 1.6 × 10^5^ U/g, was purchased from Novozymes A/S (Beijing, China). To evaluate the effect of parameters including enzyme loading, hydrolysis time and solid/liquid (S:L) ratio in enzymatic hydrolysis, a 3 × 3 × 3 factorial design was applied. The DSM was mixed with distilled water by a S:L ratio of 1:20 and 1:30 followed by protease addition of 8000, 10000 and 12000 U/g respectively. Then the enzymatic hydrolysis was conducted at 50 °C with constant agitation (100 rpm orbital shaking) for 8, 10 and 12 h. After enzymatic hydrolysis, the mixture was heated at 85 °C for 15 min to inactivate the enzyme and centrifuged at 10,000 rpm for 10 min. The supernatant separated was referred to as DSM hydrolysates which was then freeze-dried and stored at 20 °C for further use. All the experiments were conducted in triplicates.

### Determination of DH

DH was determined as the ratio of the number of broken peptide bonds to that of total bonds per unit weight which was calculated using the method of Adler-Nissen^55^:

2 $$DH \% =\frac{h\times 100}{{h}_{tot}}=\frac{B\times {N}_{b}\times 100}{\alpha \times {M}_{{\rm{p}}}\times {h}_{tot}}$$

where *h* and *h*_*tot*_ are the hydrolysis equivalents (meqv/g protein) and total number of peptide bonds in the protein substrate (7.75 meqv/g) respectively; *N*_*b*_ is normality of the base; *B* is the base consumption (ml); α is average dissociation degree of the α-NH_2_ groups and *M*_*P*_ is protein mass (g).

### Determination of the amino acids composition

The free amino acid compositions in DSM hydrolysates were determined by online pre-column derivazation RP-HPLC (Waters 600 series, Waters Corporation Milford, MA, USA). The HPLC system was equipped with a Waters 2996 Phtodiode Array Detector and a Ultimate® Amino Acid column (4.6 mm × 250 mm, 3.5 μm, Welch Materials). The mobile phase was A: 0.1 M sodium acetarsenate (pH 6.5):acetonitrile (93:7) and B: water:acetonitrile (20:80). The derivazation of the samples and the standard amino acids and determination process were recommended by Welch Materials (http://www.welch-us.com). The standard of amino acids including glutamic acid, aspartic acid, serine, histidine, glycine, methionine, arginine, alanine, threonine, proline, cysteine, tyrosine, valine, isoleucine, lysine, leucine and phenylalanine were provided by Welch Materials.

### Analysis of molecular weight distribution of peptides

The molecular weight distribution of peptides in DSM was determined by the method of Jung *et al*.^56^ with minor modifications. The samples were centrifuged at 4,000× g for 5 min. The supernatant was filtered with 4.5 μm microporous membrane. The supernatant were determined using a Waters515 type gel permeation chromatograph equipped with Ultrahydrogel water-soluble GPC column (7.8 × 3,000 mm, Waters, USA). The mobile phase was acetonitrile: water: trifluoroacetic acid = 40: 60: 0.1, added NaCl to a concentration of 0.1 M, the flow rate is 0.6 mL/min, and the molecular weight of the peptides were monitored using Waters 410 differential detector (Waters, USA) at 40 °C. The injection volume was 10 μL.

### Optimization of DSM medium

The experiment design consisted of two steps: Plactkett-Burman design (PBD)^30^ aiming to identify which fermentation parameters had a significant effect on nisin production by *L*. *lactis* F44 and central composite design (CCD)^57^ aiming to optimize these fermentation parameters.

The regression analysis of the variables was performed by using Minitab 15.0 software (Minitab Inc. Pennsylvania, USA). In the analysis, the variables which had the *p*-value less than 0.05 were considered to have a more significant impact on the response. In this study, the experimental design consisted of 13 trials as shown in Table 5, and there were 5 repetitions of the experiments at the center point. All the experiments were conducted in triplicate and the average value was recorded as the response. The behavior of the system was determined by assuming a second order polynomial function with linear, quadratic and interaction effects as follows:

3 $$Y={b}_{0}+\sum _{i=1}^{n}{b}_{i}{X}_{i}+\sum _{i=1}^{n}{b}_{ii}{X}_{i}^{2}+\sum _{i}^{n}\sum _{j}^{n}{b}_{ij}{X}_{i}{X}_{j}$$

where Y is response; X_i_ and X_j_ are independent variables; b_0_ is the offset term; b_i_ is i^th^ the linear coefficient; b_ii_ is i^th^ quadratic coefficient and b_ij_ is ij^th^ interaction coefficient.

The regression analysis of variance and preparation of response surface graphs were implemented using Design Expert software (Version 8.0.5b, State-Ease Inc., Minneapolis, USA). The optimal fermentation medium parameters for maximum nisin production by *L*. *lactis* were estimated by statistical analysis.

### Plasmids and strains construction

The primers of proteolytic system genes used in the study which were designed by primer premier 5 (Premier, Canada) are listed in Supplementary Table S2. These genes were directly amplified from *L*. *lactis* F44 or *B*. *subtilis* 168 via polymerase chain reaction (PCR). The restriction enzyme cutting sites were simultaneously inserted into the amplified gene. The resulting fragments were digested with BamHI and HindIII (or SmaI and NcoI), and then ligated into plasmid pLEB124, cut with BamHI and HindIII (or SmaI and NcoI) to generate the resulting plasmids. The resulting plasmids were transformed into *E*.*coli* TG1 by heat shock transformation for enrichment. After antibiotics selection, the plasmids were extracted with TIANprep Mini Plasmid Kit (TIANGEN, China), and then transformed into the *L*. *lactis* F44 by electroporation transformation. To construct strain BAFM, *nprB*, *oppA*, *pepF* and *pepM* were fused with the linear plasmid using the EasyGeno Assembly Cloning kit (TIANGEN, China). All the constructed plasmids were confirmed by restriction enzyme digestion and DNA sequencing.

### Fermentation experiments

The fermentation experiments with *L*. *lactis* strains were conducted in 250 ml conical flasks containing 100 ml DSM medium or commercial medium (1.5% peptone, 1.5% yeast extract, 2.0% sucrose, 2.0% KH_2_PO_4_, 0.15% NaCl, 0.3% corn steep liquor, 0.26% cysteine, and 0.015% MgSO_4_·7H_2_O) at 30 °C statically. Samples were withdraw at regular intervals and analyzed for nisin production, cell density and pH. The pH value was detected by pH meter, and cell density was measured with optical density at 600 nm. All the experiments were conducted in triplicates.

### Nisin activity assay

Nisin activity was determined by using the plate diffusion method^58^. The standardized nisin concentrate was purchased from Sigma Chemical Company (Shanghai, PR China). A stock solution of nisin was prepared by dissolving 0.1 g of nisin in 10 ml of 0.02 M HCl (10^4^ IU/ml). The solution was diluted with 0.02 M HCl to 200, 100, 50 and 25 IU/ml. Then fermentation broth (500 μL) was mixed with 500 μL of 0.02 M HCl. The mixture was boiled for 5 min and then centrifuged at 5000 rpm for 5 min at room temperature. The supernatant was appropriately diluted with 0.02 M HCl. After autoclaving, assay medium (26 ml) was cooled to about 50 °C and then inoculated with 1% (v/v) indicator strain *M*. *flavus* ATCC 10240 (the final concentration of *M*. *flavus* ATCC 10240 was 10^7^ cfu/ml). The medium was then poured into a sterile plate. After solidification, the plate was placed at 4 °C for 24 h for precultivation, which enhances nisin diffusion into the agar medium). Test wells were then bored into the assay agar plate (8 wells per plate) using a 7-mm-diameter hole punch. Standard nisin solutions and test solutions were then transferred into individual wells (100 μL per well). The plates were incubated at 30 °C and inhibition zones were measured after 24 h. Then a regression equation was calculated from the data. Each assay of standard sample or the broth sample was performed in triplicate.

### Statistical analysis

Experimental data were expressed as means ± standard deviations. SPSS 18.0 software (SPSS, Chicago, IL, USA) was applied to conduct all the statistical analyses. One-way analysis of variance (ANOVA) was performed to determine the differences for OD_600_, pH and nisin titer between control and experimental groups. A *p*-value < 0.05 was considered statistically significant.

## Electronic supplementary material

Supplementary information
