# Supplementary material for: Improving nitrogen source utilization from defatted soybean meal for nisin production by enhancing proteolytic function of Lactococcus lactis F44
Source: Sci Rep. 2017 Jul 21;7:6189. doi: 10.1038/s41598-017-06537-w (PMC5522456; doi:10.1038/s41598-017-06537-w)
Supplement: Supplementary file 1 — Supplementary information [file 41598_2017_6537_MOESM1_ESM.pdf]

Supplementary Data for:

Improving nitrogen source utilization from defatted soybean meal for nisin production by enhancing proteolytic function of *Lactococcus lactis* F44

Jiaheng Liu[1], Jianjian Zhou[1], Lihong Wang, Zelin Ma, Guangrong Zhao, Zhiqiang Ge, Hongji Zhu, Jianjun Qiao[2]

[1]These authors contributed equally to this work.

[2]Correspondence and requests for materials should be addressed to J.Q. (email: jianjunq@tju.edu.cn)

Contents:

Supplementary Tables S1-S2

Supplementary Figure S1-S2

Supplementary Table S1. Strains and plasmids used in this study.

Designation	Relevant characteristics	Sources or references
**Strains**		
Lactococcus lactis F44	nisin Z producer, constructed through genome shuffling of *L. lactis* YF11	1
Escherichia coli TG1	used for plasmid preparation	Laboratory stock
Micrococcus flavus	used as an indicator strain for bioassay of nisin	ATCC 10240
Bacillus subtilis 168	Used for cloning heterologous protease	Laboratory stock
L. lactis F44/poppA	F44 harboring pLOPPA	This study
L. lactis F44/poppB	F44 harboring pLOPPB	This study
L. lactis F44/poppC	F44 harboring pLOPPC	This study
L. lactis F44/poppD	F44 harboring pLOPPD	This study
L. lactis F44/poppF	F44 harboring pLOPPF	This study
L. lactis F44/pnprE	F44 harboring pLNPRE	This study
L. lactis F44/pnprB	F44 harboring pLNPRB	This study
L. lactis F44/pvpr	F44 harboring pLVPR	This study
L. lactis F44/ppepA	F44 harboring pLPEPA	This study
L. lactis F44/ppepF	F44 harboring pLPEPF	This study
L. lactis F44/ppepM	F44 harboring pLPEPM	This study
L. lactis F44/ppepN	F44 harboring pLPEPN	This study
L. lactis BAFM	F44 harboring pLBAFM	This study
**Plasmids**		

pLEB124	Gram^{+} cloning vector with an P45 promoter followed by multiple restriction sites, Em^{r}	2
pLOPPA	pLEB124 carrying the *oppA* gene from F44, Em^{r}	This study
pLOPPB	pLEB124 carrying the *oppB* gene from F44, Em^{r}	This study
pLOPPC	pLEB124 carrying the *oppC* gene from F44, Em^{r}	This study
pLOPPD	pLEB124 carrying the *oppD* gene from F44, Em^{r}	This study
pLOPPF	pLEB124 carrying the *oppE* gene from F44, Em^{r}	This study
pLNPRE	pLEB124 carrying the *nprE* gene from *B. subtilis* 168, Em^{r}	This study
pLNPRB	pLEB124 carrying the *nprB* gene from *B. subtilis* 168, Em^{r}	This study
pLVPR	pLEB124 carrying the *vpr* gene from *B. subtilis* 168, Em^{r}	This study
pLPEPA	pLEB124 carrying the *pepA* gene from F44, Em^{r}	This study
pLPEPF	pLEB124 carrying the *pepF* gene from F44, Em^{r}	This study
pLPEPM	pLEB124 carrying the *pepM* gene from F44, Em^{r}	This study
pLPEPN	pLEB124 carrying the *pepN* gene from F44, Em^{r}	This study
pLBAFM	pLEB124 carrying the *nprB* from *B. subtilis* 168, *oppA*, *pepF* and *pepM* gene from F44, Em^{r}	This study

Supplementary Table S2. Primers used in this study.

Primer name	Primer sequence (5 to 3’) [a,b]
nprB-F	TCC**CCCGGG**AAATGGTAGATTCCAAAATAGG
nprB-R	CATG**CCATGG**CAGTCAGACTCTGTGTCCTGA
nprE-F	CGC**GGATCC**AAAAAGGGGGATTTATTGTG
nprE-R	TCC**CCCGGG**TTACAATCCAACAGCATTCC
vpr-F	CGC**GGATCC**TTTTCTAAATACAAAGGGGGA
vpr-R	TCC**CCCGGG**ATCGCTTATTGCAGGAGG
oppA-F	CGC**GGATCC** AGTGACTCACCGATGAAAGC
oppA-R	TCC**CCCGGG** TGCACCATAGTCAAGCGTC
oppB-F	CGC**GGATCC**TAAGTCAGAGCCATTGGGC
oppB-R	TCC**CCCGGG**TCACTCCTTATTGAATCCGAA
oppC-F	CGC**GGATCC**CGGATTCAATAAGGAGTGAAAA
oppC-R	TCC**CCCGGG** GTTCGCAACGAAGTCGTTAC
oppD-F	CGC**GGATCC**TCAGGTTCTGGGAAGTCAAC
oppD-R	TCC**CCCGGG**AATCTACTCCATCAACGGCTA
oppF-F	CGC**GGATCC**TCATTTTGTTCGAGGTCAGG
oppF-R	TCC**CCCGGG**CCAATCAAACCTGAAAATGG
pepA-F	CGC**GGATCC**CGGAGGAGAAAAATGGAA
pepA-R	TCC**CCCGGG**TCCTAGTGATTTTTTCTTTTGT
pepF-F	CGC**GGATCC**TGCTAATAAGTTTTTAATGTGAAT
pepF-R	TCC**CCCGGG**TGGGGTTACTTGTACGTTTA
pepM-F	CGC**GGATCC**CTGAGTTTTTCTCAGAACTTTAGA
pepM-R	TCC**CCCGGG**CCTCTCATCTGTTTTTCCTTT
pepN-F	CGC**GGATCC**TTTAGGAGAAAACATGGC
pepN-R	TCC**CCCGGG**AAGTCAGACTAAGAATGAATAA
BAFM-pepF-F	TATATAGGGCCCGGGTGCTAATAAGTTTTTAATGTGAAT
BAFM-pepF-R	CTGAGAAAAACTCAGTGGGGTTACTTGTACGTTTA
BAFM-pepM-F	GTACAAGTAACCCCACTGAGTTTTTCTCAGAACTTTAGA
BAFM-pepM-R	TGGAATCTACCATTTCCTCTCATCTGTTTTTCCTTT
BAFM-nprB-F	AAAACAGATGAGAGGAAATGGTAGATTCCAAAATAGG
BAFM-nprB-R	CATCGGTGAGTCACTCAGTCAGACTCTGTGTCCTGA
BAFM-oppA-F	CACAGAGTCTGACTGAGTGACTCACCGATGAAAGC
BAFM-oppA-R	TAATTATAACCCGGGTGCACCATAGTCAAGCGTC

[a] Underlined and bold letters indicate restriction sites.

[b] Underlined sequences indicate homologous sequences.

Supplementary Figure and Legend

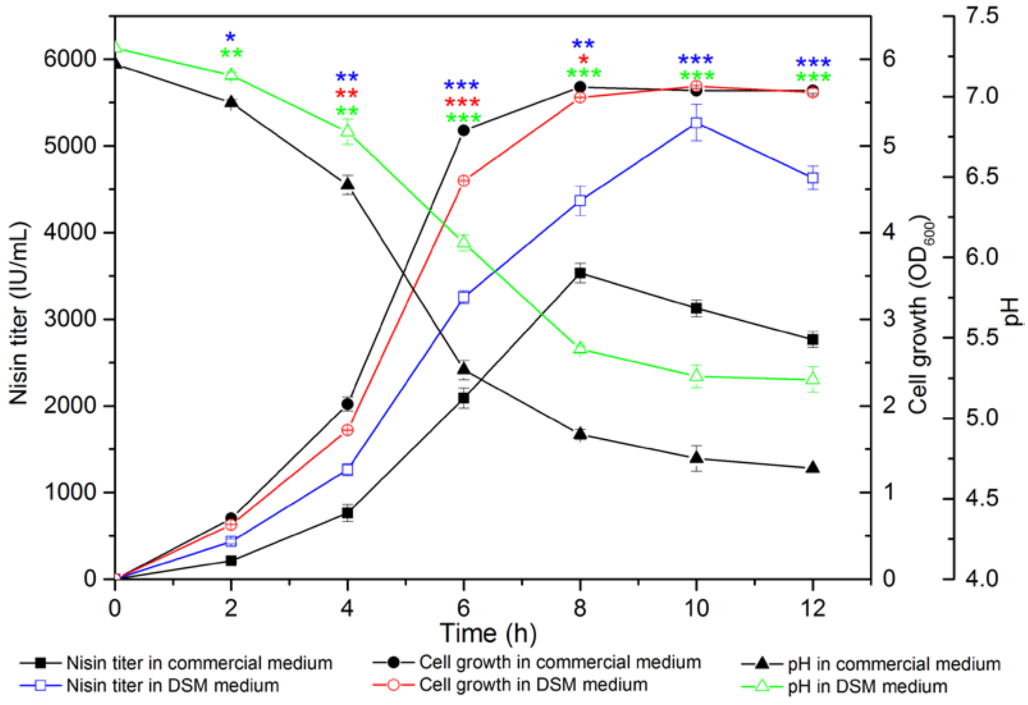

Supplementary Figure S1. Time profile of nisin titer (square), cell density (circle) and pH (triangle) of *L. lactis* BAFM cultured in DSM medium with 21 g/L DSM hydrolysates (hollow symbols) and commercial medium (solid symbols). Average data of triplicate experiments were presented. Error bars represent standard deviations from three parallel replicates. The data were analyzed by One-way ANOVA. Statistically significant differences between DSM medium groups and commercial medium groups were indicated by * for $p < 0.05$ ** for $p < 0.01$ and *** for $p < 0.001$.

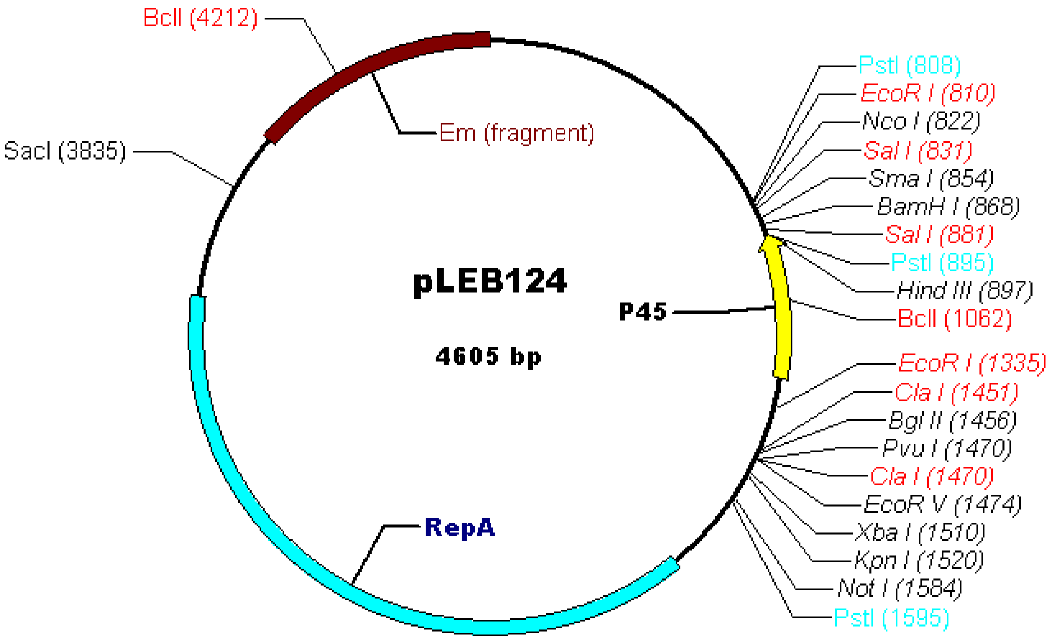

<u>Supplementary Figure</u> S2. Physical map of pLEB124.

References

1. Zhang, Y. F. *et al*. Genome shuffling of *Lactococcus lactis subspecies lactis* YF11 for improving nisin Z production and comparative analysis. *J Dairy Sci*. **97**, 2528-2541 (2014).
2. Qiao M, Immonen T, Koponen O. & Saris P. E. J. The cellular location and effect on nisin immunity of the NisI protein from *Lactococcus lactis* N8 expressed in *Escherichia coli* and *L. lactis*. *FEMS Microbiol. Lett.* 131:75–80 (1995).
